# 2-[(4-Methyl­benzo­yl)hydrazono]­propionic acid monohydrate

**DOI:** 10.1107/S1600536809003067

**Published:** 2009-01-31

**Authors:** Hon Wee Wong, Kong Mun Lo, Seik Weng Ng

**Affiliations:** a Department of Chemistry, University of Malaya, 50603 Kuala Lumpur, Malaysia

## Abstract

In the title compound, C_11_H_12_N_2_O_3_·H_2_O, the water mol­ecule is a hydrogen-bond donor to the double-bond amide and the carbonyl O atoms of two acid mol­ecules; it is also a hydrogen-bond acceptor to the acid –OH and amide –NH– groups. These hydrogen-bonding inter­actions give rise to a layer structure, with the layers parallel to the *ab* plane.

## Related literature

The deprotonated anion of 2-aroylhydrazonopropionic acid furnishes a number of metal complexes; see, for example: Wu, Chen *et al.* (2006 ▶); Liu *et al.* (2007 ▶); Wu & Zeng (2007 ▶); Wu *et al.* (2006*a* ▶,*b* ▶); Yang *et al.* (2004 ▶); Yin & Chen (2006 ▶); Zhai *et al.* (2007 ▶).
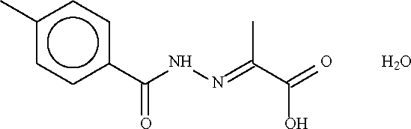


## Experimental

### 

#### Crystal data

C_11_H_12_N_2_O_3_·H_2_O
*M* *_r_* = 238.24
Monoclinic, 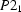

*a* = 6.8464 (1) Å
*b* = 11.9753 (2) Å
*c* = 7.0005 (1) Å
β = 102.169 (1)°
*V* = 561.06 (2) Å^3^
*Z* = 2
Mo *K*α radiation
μ = 0.11 mm^−1^
*T* = 100 (2) K
0.20 × 0.10 × 0.10 mm

#### Data collection

Bruker SMART APEX diffractometer
Absorption correction: none
5272 measured reflections
1335 independent reflections
1211 reflections with *I* > 2σ(*I*)
*R*_int_ = 0.029

#### Refinement

*R*[*F* ^2^ > 2σ(*F* ^2^)] = 0.030
*wR*(*F* ^2^) = 0.083
*S* = 1.02
1335 reflections
172 parameters
5 restraints
H atoms treated by a mixture of independent and constrained refinement
Δρ_max_ = 0.19 e Å^−3^
Δρ_min_ = −0.16 e Å^−3^
Absolute structure: 1126 Friedel pairs were merged

### 

Data collection: *APEX2* (Bruker, 2007 ▶); cell refinement: *APEX2*; data reduction: *SAINT* (Bruker, 2007 ▶); program(s) used to solve structure: *SHELXS97* (Sheldrick, 2008 ▶); program(s) used to refine structure: *SHELXL97* (Sheldrick, 2008 ▶); molecular graphics: *X-SEED* (Barbour, 2001 ▶); software used to prepare material for publication: *publCIF* (Westrip, 2009 ▶).

## Supplementary Material

Crystal structure: contains datablocks global, I. DOI: 10.1107/S1600536809003067/cv2513sup1.cif

Structure factors: contains datablocks I. DOI: 10.1107/S1600536809003067/cv2513Isup2.hkl

Additional supplementary materials:  crystallographic information; 3D view; checkCIF report

## Figures and Tables

**Table 1:** Hydrogen-bond geometry (Å, °)

*D*—H⋯*A*	*D*—H	H⋯*A*	*D*⋯*A*	*D*—H⋯*A*
O1—H1⋯O1*W*	0.83 (2)	2.03 (2)	2.777 (2)	149 (3)
O1*W*—H11⋯O3	0.84 (2)	1.97 (2)	2.794 (2)	165 (4)
O1*W*—H12⋯O2^i^	0.84 (2)	2.00 (1)	2.829 (2)	168 (3)
N2—H2⋯O1*W*^ii^	0.87 (2)	2.35 (1)	3.210 (2)	168 (3)

Symmetry codes: (i) 
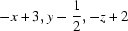
; (ii) 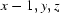.

**Fig. 1.**
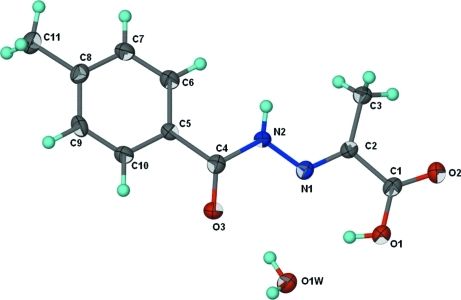
Displacement ellipsoids plot (Barbour, 2001) of the title compound at the 70% probability level. Hydrogen atoms are drawn as spheres of arbitrary radius.

## supplementary crystallographic information

### Experimental

4-Toluihydrazide (1 g, 0.007 mol) and pyruvic acid (0.6 g, 0.007 mol) were dissolved in methanol (30 ml). The solution was heated for 3 h; slow evaporation of the solvent gave colorless crystals.

### Refinement

Carbon-bound H atoms were placed in calculated positions (C—H 0.93–0.99 Å) and were included in the refinement in the riding model approximation, with *U*(H) set to 1.2 to 1.5*U*(C). The methyl H atoms were rotated to fit the electron density.

The oxygen- and nitrogen-bound H atoms were located in a difference Fourier map, and were refined with distance restraints [N—H 0.88 (2) and O—H 0.84 (2) Å]; their temperature factors were freely refined.

### Crystal data

**Table:**

C_11_H_12_N_2_O_3_·H_2_O	*F*(000) = 252
*M**_r_* = 238.24	*D*_x_ = 1.410 Mg m^−^^3^
Monoclinic, *P*2_1_	Mo *K*α radiation, λ = 0.71073 Å
Hall symbol: P 2yb	Cell parameters from 1743 reflections
*a* = 6.8464 (1) Å	θ = 3.0–26.9°
*b* = 11.9753 (2) Å	µ = 0.11 mm^−^^1^
*c* = 7.0005 (1) Å	*T* = 100 K
β = 102.169 (1)°	Irregular block, colourless
*V* = 561.06 (2) Å^3^	0.20 × 0.10 × 0.10 mm
*Z* = 2	

### Data collection

**Table:**

Bruker SMART APEX diffractometer	1211 reflections with *I* > 2σ(*I*)
Radiation source: fine-focus sealed tube	*R*_int_ = 0.029
graphite	θ_max_ = 27.5°, θ_min_ = 3.0°
ω scans	*h* = −8→8
5272 measured reflections	*k* = −14→15
1335 independent reflections	*l* = −9→9

### Refinement

**Table:**

Refinement on *F*^2^	Secondary atom site location: difference Fourier map
Least-squares matrix: full	Hydrogen site location: inferred from neighbouring sites
*R*[*F*^2^ > 2σ(*F*^2^)] = 0.030	H atoms treated by a mixture of independent and constrained refinement
*wR*(*F*^2^) = 0.083	*w* = 1/[σ^2^(*F*_o_^2^) + (0.0559*P*)^2^ + 0.0248*P*] where *P* = (*F*_o_^2^ + 2*F*_c_^2^)/3
*S* = 1.02	(Δ/σ)_max_ = 0.001
1335 reflections	Δρ_max_ = 0.19 e Å^−^^3^
172 parameters	Δρ_min_ = −0.16 e Å^−^^3^
5 restraints	Absolute structure: 1126 Friedel pairs were merged
Primary atom site location: structure-invariant direct methods	

### Special details

**Table:**

Geometry. All e.s.d.'s (except the e.s.d. in the dihedral angle between two l.s. planes) are estimated using the full covariance matrix. The cell e.s.d.'s are taken into account individually in the estimation of e.s.d.'s in distances, angles and torsion angles; correlations between e.s.d.'s in cell parameters are only used when they are defined by crystal symmetry. An approximate (isotropic) treatment of cell e.s.d.'s is used for estimating e.s.d.'s involving l.s. planes.

### Fractional atomic coordinates and isotropic or equivalent isotropic displacement parameters (Å^2^)

**Table:**

	*x*	*y*	*z*	*U*_iso_*/*U*_eq_	
O1	1.4450 (2)	0.99988 (13)	0.8798 (3)	0.0255 (4)	
O2	1.2160 (2)	1.13156 (13)	0.8463 (2)	0.0269 (4)	
O3	1.2831 (2)	0.64501 (13)	0.7200 (2)	0.0260 (4)	
O1W	1.6032 (2)	0.78718 (13)	0.8663 (3)	0.0251 (4)	
N1	1.1624 (2)	0.84748 (15)	0.7821 (3)	0.0192 (4)	
N2	1.0279 (3)	0.76060 (15)	0.7458 (3)	0.0202 (4)	
C1	1.2584 (3)	1.03349 (18)	0.8395 (3)	0.0204 (5)	
C2	1.0961 (3)	0.94692 (18)	0.7861 (3)	0.0183 (4)	
C3	0.8847 (3)	0.98625 (18)	0.7447 (3)	0.0232 (5)	
H3A	0.8131	0.9412	0.6686	0.035*	
H3B	0.8400	0.9951	0.8651	0.035*	
H3C	0.8754	1.0568	0.6777	0.035*	
C4	1.1067 (3)	0.65752 (18)	0.7236 (3)	0.0198 (4)	
C5	0.9684 (3)	0.56054 (18)	0.7054 (3)	0.0178 (4)	
C6	0.7608 (3)	0.5709 (2)	0.6510 (3)	0.0218 (4)	
H6	0.7018	0.6422	0.6192	0.026*	
C7	0.6408 (3)	0.47675 (19)	0.6437 (3)	0.0231 (5)	
H7	0.4998	0.4845	0.6067	0.028*	
C8	0.7226 (3)	0.37199 (18)	0.6891 (3)	0.0214 (5)	
C9	0.9315 (3)	0.3617 (2)	0.7379 (3)	0.0221 (5)	
H9A	0.9904	0.2901	0.7659	0.026*	
C10	1.0524 (3)	0.45480 (19)	0.7457 (3)	0.0202 (4)	
H10	1.1936	0.4467	0.7787	0.024*	
C11	0.5927 (3)	0.27036 (19)	0.6870 (4)	0.0288 (5)	
H11A	0.6157	0.2411	0.7985	0.043*	
H11B	0.4542	0.2924	0.6689	0.043*	
H11C	0.6071	0.2211	0.5815	0.043*	
H11	1.496 (3)	0.755 (3)	0.813 (5)	0.059 (10)*	
H12	1.653 (4)	0.748 (2)	0.963 (3)	0.052 (10)*	
H1	1.445 (5)	0.9307 (9)	0.867 (5)	0.056 (11)*	
H2	0.906 (2)	0.773 (2)	0.761 (4)	0.031 (7)*	

### Atomic displacement parameters (Å^2^)

**Table:**

	*U*^11^	*U*^22^	*U*^33^	*U*^12^	*U*^13^	*U*^23^
O1	0.0193 (8)	0.0198 (9)	0.0371 (10)	−0.0029 (6)	0.0049 (7)	−0.0031 (7)
O2	0.0234 (7)	0.0182 (8)	0.0384 (9)	−0.0001 (6)	0.0047 (7)	−0.0028 (7)
O3	0.0171 (7)	0.0206 (8)	0.0416 (9)	−0.0005 (6)	0.0094 (6)	−0.0039 (7)
O1W	0.0205 (7)	0.0208 (8)	0.0331 (10)	−0.0018 (6)	0.0036 (7)	0.0028 (7)
N1	0.0186 (9)	0.0171 (9)	0.0218 (9)	−0.0017 (7)	0.0042 (7)	0.0008 (7)
N2	0.0139 (8)	0.0165 (9)	0.0301 (10)	−0.0001 (7)	0.0048 (7)	−0.0012 (7)
C1	0.0198 (10)	0.0212 (11)	0.0202 (11)	−0.0033 (8)	0.0043 (8)	−0.0013 (8)
C2	0.0167 (9)	0.0179 (10)	0.0209 (10)	−0.0012 (8)	0.0054 (8)	−0.0004 (8)
C3	0.0174 (10)	0.0189 (11)	0.0329 (13)	0.0007 (8)	0.0044 (9)	0.0033 (9)
C4	0.0200 (10)	0.0186 (10)	0.0207 (10)	−0.0004 (8)	0.0041 (8)	0.0004 (9)
C5	0.0170 (10)	0.0169 (10)	0.0201 (10)	0.0010 (8)	0.0049 (8)	−0.0013 (8)
C6	0.0205 (10)	0.0204 (10)	0.0240 (11)	0.0024 (9)	0.0036 (8)	−0.0003 (9)
C7	0.0159 (10)	0.0238 (11)	0.0289 (12)	0.0017 (9)	0.0034 (8)	−0.0049 (9)
C8	0.0225 (11)	0.0216 (11)	0.0211 (11)	−0.0046 (9)	0.0068 (8)	−0.0057 (9)
C9	0.0260 (11)	0.0154 (10)	0.0254 (11)	0.0042 (9)	0.0067 (9)	0.0005 (8)
C10	0.0160 (9)	0.0214 (11)	0.0226 (11)	0.0021 (8)	0.0030 (8)	−0.0004 (9)
C11	0.0282 (11)	0.0228 (12)	0.0370 (14)	−0.0050 (10)	0.0110 (10)	−0.0044 (10)

### Geometric parameters (Å, °)

**Table:**

O1—C1	1.312 (3)	C4—C5	1.487 (3)
O1—H1	0.83 (2)	C5—C10	1.395 (3)
O2—C1	1.213 (3)	C5—C6	1.397 (3)
O3—C4	1.222 (2)	C6—C7	1.390 (3)
O1W—H11	0.84 (2)	C6—H6	0.9500
O1W—H12	0.82 (2)	C7—C8	1.383 (3)
N1—C2	1.277 (3)	C7—H7	0.9500
N1—N2	1.377 (2)	C8—C9	1.404 (3)
N2—C4	1.369 (3)	C8—C11	1.506 (3)
N2—H2	0.87 (2)	C9—C10	1.383 (3)
C1—C2	1.508 (3)	C9—H9A	0.9500
C2—C3	1.491 (3)	C10—H10	0.9500
C3—H3A	0.8400	C11—H11A	0.8400
C3—H3B	0.9620	C11—H11B	0.9663
C3—H3C	0.9620	C11—H11C	0.9662
			
C1—O1—H1	108 (2)	C6—C5—C4	123.14 (19)
H11—O1W—H12	106 (3)	C7—C6—C5	119.8 (2)
C2—N1—N2	118.80 (17)	C7—C6—H6	120.1
C4—N2—N1	116.00 (17)	C5—C6—H6	120.1
C4—N2—H2	124.9 (19)	C8—C7—C6	121.34 (19)
N1—N2—H2	118.1 (19)	C8—C7—H7	119.3
O2—C1—O1	121.2 (2)	C6—C7—H7	119.3
O2—C1—C2	120.35 (19)	C7—C8—C9	118.5 (2)
O1—C1—C2	118.40 (18)	C7—C8—C11	121.41 (19)
N1—C2—C3	128.72 (19)	C9—C8—C11	120.1 (2)
N1—C2—C1	113.55 (17)	C10—C9—C8	120.6 (2)
C3—C2—C1	117.73 (19)	C10—C9—H9A	119.7
C2—C3—H3A	109.5	C8—C9—H9A	119.7
C2—C3—H3B	109.9	C9—C10—C5	120.40 (18)
H3A—C3—H3B	112.0	C9—C10—H10	119.8
C2—C3—H3C	109.5	C5—C10—H10	119.8
H3A—C3—H3C	106.6	C8—C11—H11A	109.5
H3B—C3—H3C	109.3	C8—C11—H11B	110.0
O3—C4—N2	121.82 (19)	H11A—C11—H11B	102.8
O3—C4—C5	121.12 (19)	C8—C11—H11C	110.2
N2—C4—C5	117.06 (17)	H11A—C11—H11C	115.2
C10—C5—C6	119.3 (2)	H11B—C11—H11C	108.8
C10—C5—C4	117.61 (17)		
			
C2—N1—N2—C4	173.5 (2)	N2—C4—C5—C6	20.0 (3)
N2—N1—C2—C3	−3.9 (3)	C10—C5—C6—C7	2.0 (3)
N2—N1—C2—C1	176.43 (17)	C4—C5—C6—C7	−177.78 (19)
O2—C1—C2—N1	179.1 (2)	C5—C6—C7—C8	−0.1 (3)
O1—C1—C2—N1	−1.5 (3)	C6—C7—C8—C9	−1.8 (3)
O2—C1—C2—C3	−0.6 (3)	C6—C7—C8—C11	178.2 (2)
O1—C1—C2—C3	178.78 (19)	C7—C8—C9—C10	1.8 (3)
N1—N2—C4—O3	−5.9 (3)	C11—C8—C9—C10	−178.2 (2)
N1—N2—C4—C5	173.67 (17)	C8—C9—C10—C5	0.1 (3)
O3—C4—C5—C10	19.8 (3)	C6—C5—C10—C9	−2.0 (3)
N2—C4—C5—C10	−159.73 (19)	C4—C5—C10—C9	177.77 (19)
O3—C4—C5—C6	−160.4 (2)		

### Hydrogen-bond geometry (Å, °)

**Table:**

*D*—H···*A*	*D*—H	H···*A*	*D*···*A*	*D*—H···*A*
O1—H1···O1W	0.83 (2)	2.03 (2)	2.777 (2)	149 (3)
O1W—H11···O3	0.84 (2)	1.97 (2)	2.794 (2)	165 (4)
O1W—H12···O2^i^	0.84 (2)	2.00 (1)	2.829 (2)	168 (3)
N2—H2···O1W^ii^	0.87 (2)	2.35 (1)	3.210 (2)	168 (3)

Symmetry codes: (i) −*x*+3, *y*−1/2, −*z*+2; (ii) *x*−1, *y*, *z*.
